# Simultaneous Activation of Kras and Inactivation of p53 Induces Soft Tissue Sarcoma and Bladder Urothelial Hyperplasia

**DOI:** 10.1371/journal.pone.0074809

**Published:** 2013-09-18

**Authors:** Xiaoping Yang, Francisco G. La Rosa, Elizabeth Erin Genova, Kendra Huber, Jerome Schaack, James DeGregori, Natalie J. Serkova, Yuan Li, Lih-Jen Su, Elizabeth Kessler, Thomas W. Flaig

**Affiliations:** 1 Division of Medical Oncology, Department of Medicine, School of Medicine, University of Colorado Anschutz Medical Campus, Aurora, Colorado, United States of America; 2 Department of Pathology, School of Medicine, University of Colorado Anschutz Medical Campus, Aurora, Colorado, United States of America; 3 Department of Anesthesiology, School of Medicine, University of Colorado Anschutz Medical Campus, Aurora, Colorado, United States of America; 4 Department of Microbiology, School of Medicine, University of Colorado Anschutz Medical Campus, Aurora, Colorado, United States of America; 5 Department of Biochemistry and Molecular Genetics, School of Medicine, University of Colorado Anschutz Medical Campus, Aurora, Colorado, United States of America; 6 University of Colorado Cancer Center, Aurora, Colorado, United States of America; Virginia Commonwealth University, United States of America

## Abstract

The development of the Cre recombinase-controlled (Cre/LoxP) technique allows the manipulation of specific tumorigenic genes, temporarily and spatially. Our original intention of this study was to investigate the role of Kras and p53 in the development of urinary bladder cancer. First, to validate the effect of intravesical delivery on Cre recombination (Adeno-Cre), we examined activity and expression of β-galactosidase in the bladder of control ROSA transgenic mice. The results confirmed specific recombination as evidenced by β-galactosidase activity in the bladder urothelium of these mice. Then, we administered the same adenovirus into the bladder of double transgenic Kras^LSLG12D/+^. p53^fl/fl^ mice. The virus solution was held in place by a distal urethral retention suture for 2 hours. To our surprise, there was a rapid development of a spindle-cell tumor with sarcoma characteristics near the suture site, within the pelvic area but outside the urinary track. Since we did not see any detectable β-galactosidase in the area outside of the bladder in the validating (control) experiment, we interpreted that this sarcoma formation was likely due to transduction by Adeno-Cre in the soft tissue of the suture site. To avoid the loss of skin integrity associated with the retention suture, we transitioned to an alternative technique without suture to retain the Adeno-Cre into the bladder cavity. Interestingly, although multiple Adeno-Cre treatments were applied, only urothelial hyperplasia but not carcinogenesis was observed in the subsequent experiments of up to 6 months. In conclusion, we observed that the simultaneous inactivation of p53 and activation of Kras induces quick formation of spindle-cell sarcoma in the soft tissues adjacent to the bladder but slow formation of urothelial hyperplasia inside the bladder. These results strongly suggest that the effect of oncogene regulation to produce either hyperplasia or carcinogenesis greatly depends on the tissue type.

## Introduction

Kras, a well-known oncogene, and p53, a notable tumor suppressor gene, are two well-studied tumorigenic genes that have been associated with lung [1,2] [3], pancreas [4,5] [6] and colon cancer [7]. However, the role of these two genes in the development of urothelial carcinoma is not well defined in in-vivo models.

p53 is a nuclear phosphoprotein that plays a central role in controlling cell growth, and its mutations are commonly observed in high-grade urothelial carcinoma [8,9,10]. Interestingly, p53 dysfunction is not common in non-invasive, superficial urothelial tumors, suggesting a role for p53 mutations in promoting tumor invasion [11,12]. In mouse models, it has been shown that p53 deficiency by itself predisposes the urothelium to proliferate, but this alone is not sufficient for bladder tumorigenesis [13]. On the other hand, the loss of p53 in the context of simultaneously activated Hras is sufficient to promote urothelial tumorigenesis [13].

Previous investigations have reported 4 to 29% in incidence rate of Kras mutations in human urothelial carcinomas [14,15,16]. However, the role of Kras in urinary bladder cancer development has not been widely examined. Recently, several pioneering and elegantly designed studies have begun to address its role in this respect [17,18]. Kras mutations may cooperate with β-catenin activation to induce urothelial cell carcinoma [18], consistent with our broad understanding of the Knudson “multiple-hit” hypothesis of tumorigenesis [19]. Furthermore, the mutant Kras induces neoplastic changes in a wide variety of tumor types [20], including squamous cell carcinoma of the oral cavity [21] and skin cancer [22].

Conditional gene targeting using the Cre/loxP system has become a useful method of studying gene function [23]. The manipulation of tumor suppressor genes and oncogenes may be controlled by the delivery of Cre recombinase to specific cells [24]. This is achieved by engineering LoxP DNA elements into the mouse genome that either surround (“Flox”) exons critical to a tumor suppressor gene’s function or around a synthetic “stop” element (“LSL”) inserted in front of an oncogene.

Cre activation of cells may occur via three distinct methods to induce modulations of genes of interest. Firstly, transgenic mice with tissue-specific Cre expression (e.g. bladder-specific gene uroplakin II promoter - UPII-Cre) have been developed to investigate the roles of genes in specific organs [25]. The limitations of this strategy include the need to have one more cross breeding of UPII-Cre and the inability to control the temporal activation of Cre. Another major disadvantage of transgenic (Tg) expression of Cre is that this promoter is expressed in all cells, which is far from the physiological context of rare activation of an oncogene in a few cells.

The second method is based on chemically-induced forms of Cre (e.g., tamoxifen) in diverse organs, including urinary bladder, for temporal gene activation or inactivation [21,26,27,28]. The strength of this method is that it is easy to apply but it requires the development of novel transgenic animals with specific receptors (i.e., Cre-estrogen receptors).

Recently, a more convenient method has been developed, which uses a recombinant adenovirus expressing Cre recombinase (Adeno-Cre) to directly manipulate the target genes [1]. One report has shown that surgical delivery of Adeno-Cre into the bladder cavity of adult male mice can be used to induce conditional gene deletion in the epithelium by the lower midline incision [29]. They were able to manipulate gene deletion exclusively in the epithelium but not in the underlying lamina propria or muscle layers. In our study, we apply this approach using urinary catheterizing to simultaneously modulate Kras and p53 genes in the mouse urinary bladder via intravesical instillation of Adeno-Cre.

## Results

### Validation of Cre-recombination activation in intravesically treated ROSA26 mice

**Figure 1:**
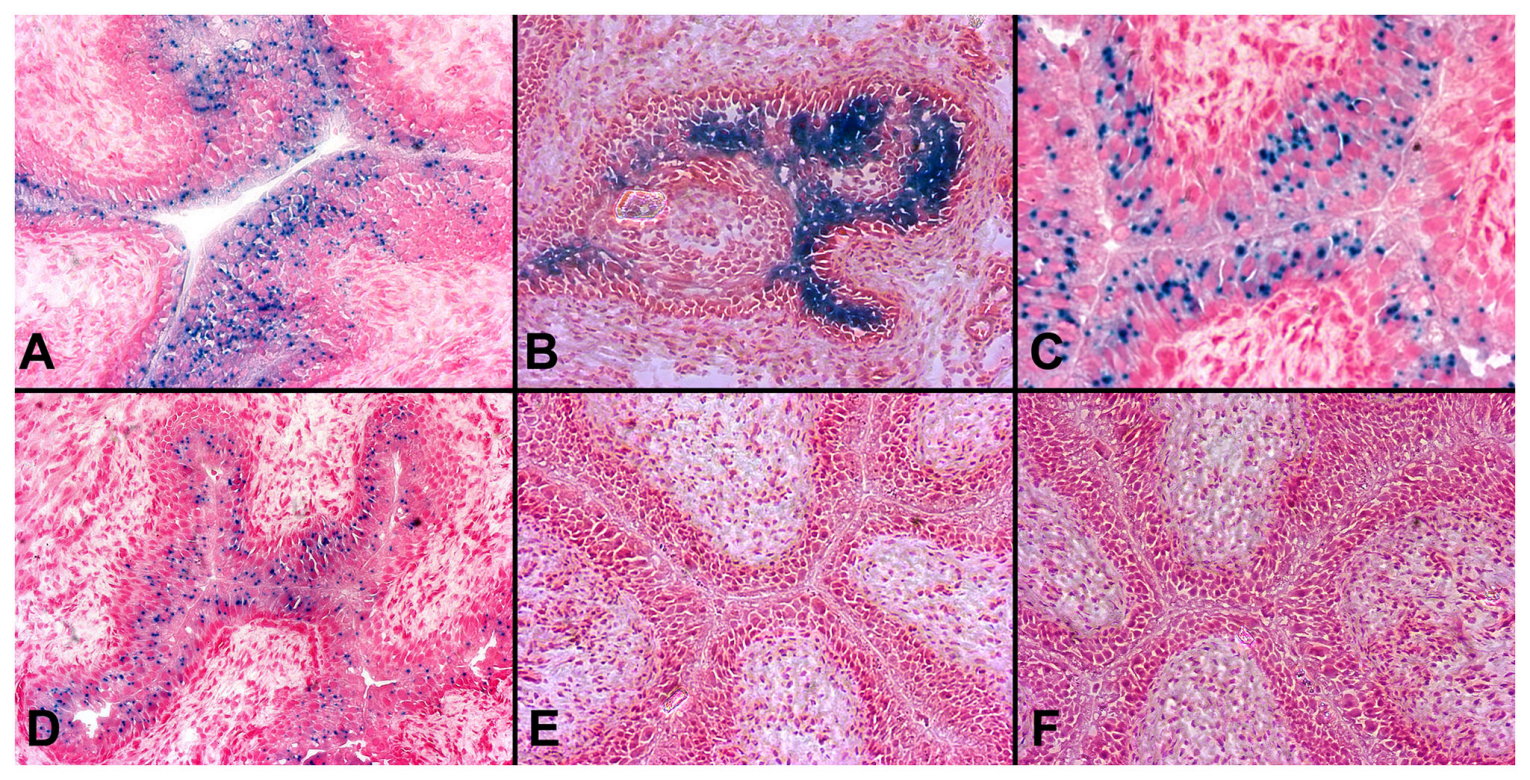
β-galactosidase staining (Blue) of urinary bladders after intravesical treatment of Adeno-Cre or Adeno-empty vector. Adeno-Cre (**A**-**C**, **F**) or Adeno-empty vector virus (**E**) (8.46×10^8^ pfu per mouse) was delivered into the bladder lumen of ROSA mice (**A**-**C**, **E**) or C57 mice (**F**) for 2 hours. The mice were sacrificed at days 3 (A), 7 (**B**), or 14 (**C**) post Adeno-Cre bladder instillation. The bladders were removed and stained for β-galactosidase (blue) expression (counterstaining with fast red). A dynamic change of β-galactosidase is seen with the maximum expression at 7 days post Adeno-Cre instillation (**B**), predominantly in the upper (inner) 2/3 layer of the urothelium. β-galactosidase activity was reduced when half of the Adeno-Cre dose (4.23 ×10^8^ pfu) (**D**) was used as compared with the full dose (**B**). Instillation of either Adeno-Cre-empty into ROSA mice (**E**) or Adeno-Cre to C57 mice (**F**) did not show β-galactosidase expression. n=5 for each group. All figures are at 10x magnification.

To characterize the *in vivo* changes and Cre activity after intravesical Adeno-Cre instillation, a reporter mouse strain ROSA26, generated by Soriano, was used as a control system [30]. Adeno-Cre was instilled into the mouse urinary bladder through the urethra and X-gal staining was used to detect β-galactosidase activity, defining the Cre-recombination effect in the mouse urothelium. Figure 1A~D show stained sections obtained from urinary bladder tissues, ranging from 3 days to 2 weeks post-Adeno-Cre transduction. Interestingly, as shown in these figures, the dynamic change of β-galactosidase activity peaked at day 7 after a single intravesical administration of Adeno-Cre (Figure 1B) and eventually faded over the next two (Figure 1C) to four weeks (image not shown). The level of β-galactosidase activity observed was dose dependent, as demonstrated by the administration of a half dose of Adeno-Cre (4.23×10^8^ pfu per mouse) (Figure 1D) compared to the dose of 8.46×10^8^ pfu per mouse post 7 days (Figure 1B). We did not use higher doses than 8.46×10^8^ pfu per mouse, since a higher dose is not available, and the instillation volume could not be further increased due to limit of mouse bladder size. These results revealed that Adeno-Cre was able to induce β-galactosidase activity in the urothelium of ROSA mice, confirming the effectiveness of this intravesical administration.

To further determine the specificity of this Cre-recombination, a negative control (adenovirus-empty vector) was instilled into the ROSA mice and we observed no expression of β-galactosidase activity (Figure 1E) at day 7. Furthermore, Adeno-Cre was also instilled into the urinary bladder of C57 mice and we found no expression of β-galactosidase activity in the bladder tissues (Figure 1F) at day 7. These negative control results support the specificity of Cre activation of the ROSA reporter genes via the intravesical delivery method.

**Figure 2:**
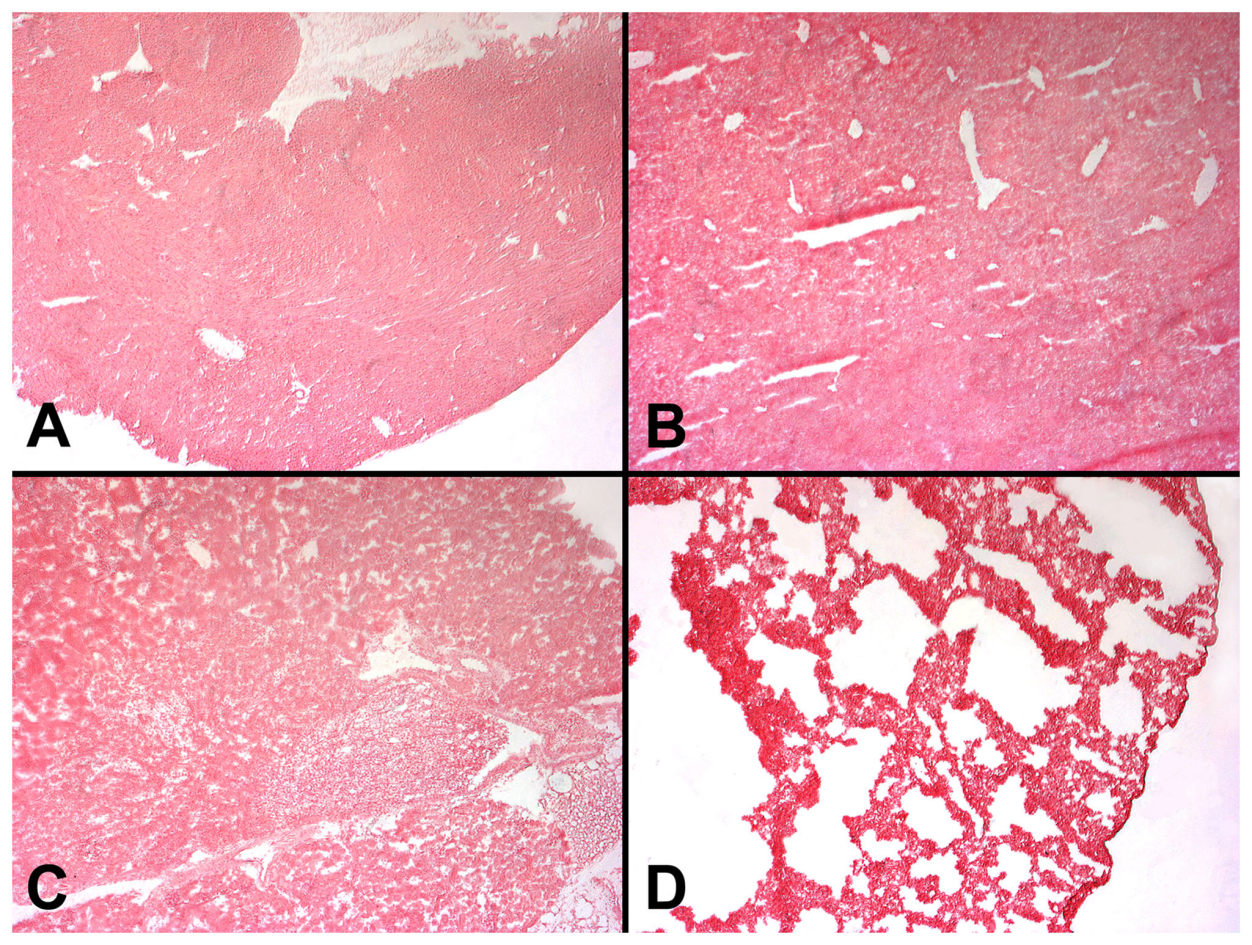
β-galactosidase staining of heart, liver, kidney and lung after intravesical treatment of Adeno-Cre. Adeno-Cre (8.46×10^8^ pfu per mouse) was delivered into the bladder lumen of ROSA mice for 2 hours (n=5). At day 7, mice were sacrificed, followed by removal of heart (**A**), liver (**B**), kidney (**C**) and lung (**D**). X-Gal staining for β-galactosidase activity was performed in all these tissues. No observable staining (blue color) was observed (only counterstaining with fast red was present), as compared with Figure 1 (A-D). All figures are at 10x magnification.

To explore the specific spatial effect of intravesical delivery of Adeno-Cre, β-galactosidase expression was also studied in other organs of the ROSA mice. In contrast to the observed specific urothelial expression in these mice (Figure 1B), we observed no expression of β-galactosidase in these organs including heart (Figure 2A), liver (Figure 2B), kidney (Figure 2C), lung **(** Figure 2D) and brain (image not shown).

**Figure 3:**
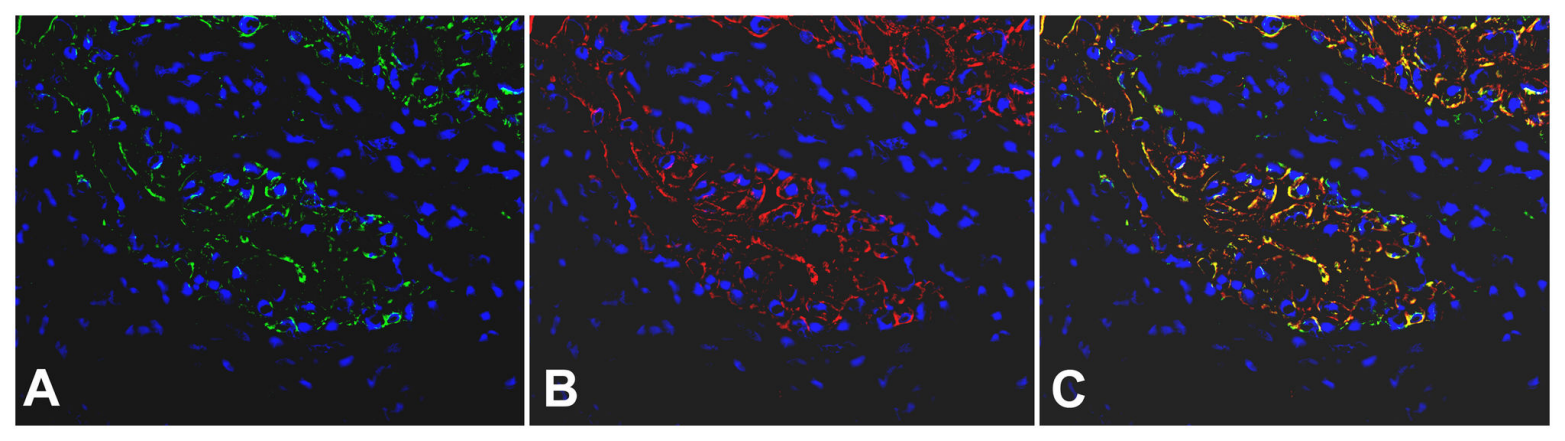
Immunofluorescent staining of β-galactosideasae and cytokeratin expression of urinary bladder after intravesical treatment with Adeno-Cre. Adeno-Cre (8.46×10^8^ pfu per mouse) was delivered into the bladder lumen of ROSA mice for 2 hours (n=5). Mice were sacrificed and their bladders were removed and processed for immunofluorescence staining. The urothelial mucosa shows in green β-galactosidase (**A**) and in red cytokeratin 7 expressions (**B**). In Figure (C), overlapping of the fields (**A**) and (**B**) demonstrates the co-localization of β-galactosidase and cytokeratin 7 expressions (orange-yellow). Nuclei were stained with DAPI (blue color). β-galactosidase expression is scattered and observed mainly in the 2/3 upper (inner) layer of the urothelium versus cytokeratin expression which is evenly present in the full thickness of the urothelium.

To further define the spatial effect of Adeno-Cre via this delivery route, we performed immunofluorescence studies on urinary bladders from the same mice and found marked β-galactosidase expression as shown in Figure 3A in green. In these same procedures the urothelium is identified in red color by the simultaneous staining with rhodamine-labelled anti-cytokeratin 7 antibody (Figure 3B). The overlapping of these two colors (orange) further demonstrated that β-galactosidase expression is specifically located within the urothelial cells only (Figure 3C). β-galactosidase expression is scattered, but observed mainly in the 2/3 upper (inner) layer of the urothelium as opposed to the cytokeratin expression, which is evenly present in the full thickness of the urothelium. These results strongly support the spatial specificity of the Adeno-Cre effect and the effectiveness of the intravesical administration route.

### Breeding and Genotyping of Kras ^LSLG12D/+^. p53 ^fl/fl^ mice

We next cross-bred B6.129S4-*Kras* ^tm4Tyj^/J and B6.129P2-*Trp53* ^tm1Brn^/J transgenic mice. B6.129S4-*Kras* ^tm4Tyj^/J mice have the point mutation G12D in the Kras gene. Expression of the mutated gene is blocked by the presence of a LoxP-flanked stop codon. Since the homozygous mutation is lethal in utero, only mice with the heterozygous Kras^LSLG12D/+^ genotype mice survive. Cre-mediated recombination of the heterozygous Kras^LSLG12D/+^ mice mediates excision of the stop codon and permits the expression of the oncogenic protein Kras [31] [20].

**Figure 4:**
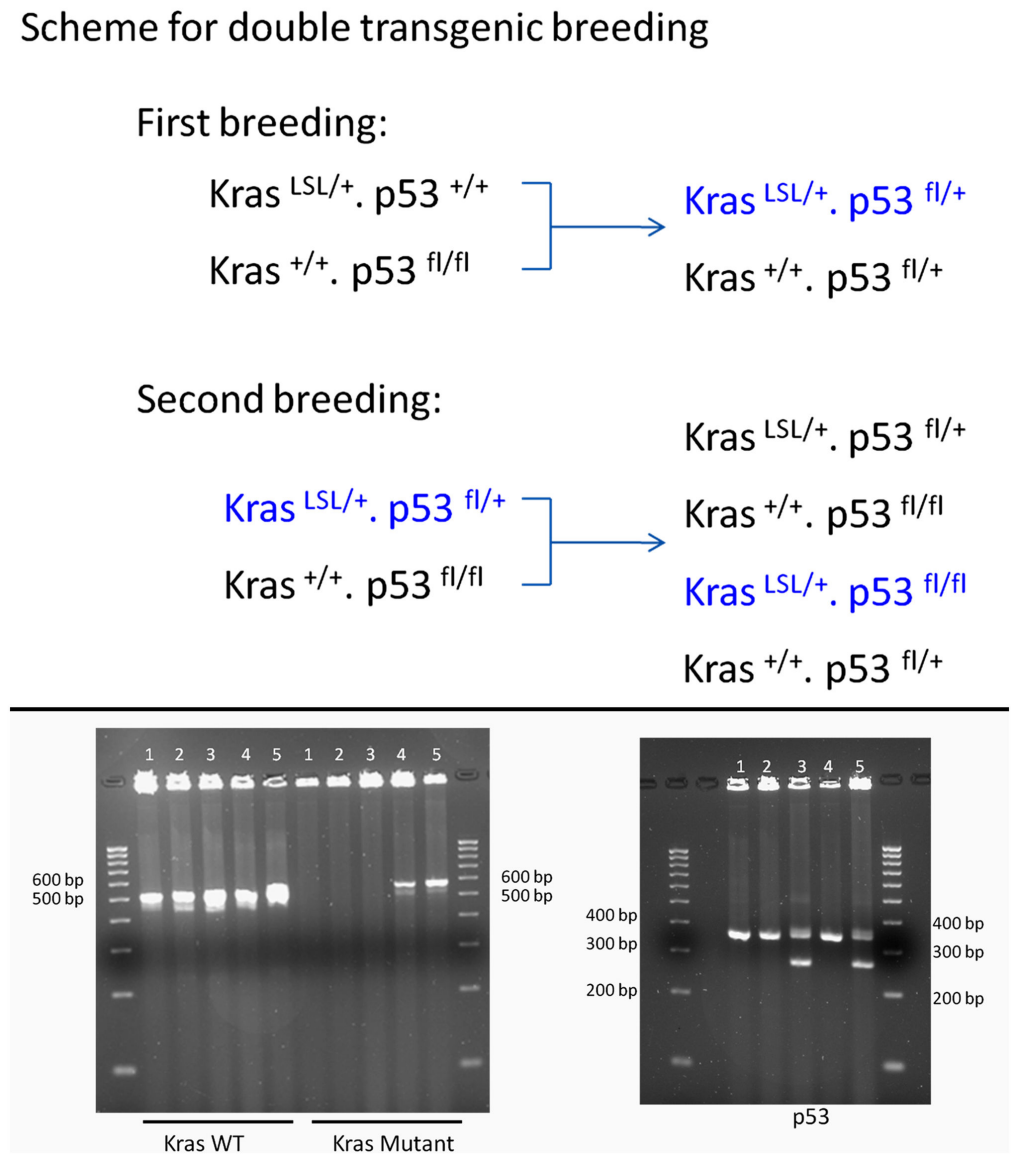
Breeding scheme and genotyping of double transgenic mice. Two-step plan for breeding double transgenic mice. **Upper**: Scheme for breeding plan starting from Kras^LSLG12D/+^. p53^+/+^ and Kras^+/+^. p53^fl/fl^. Useful mice are marked in blue text. **Lower**: Agarose gel imaging for genotyping: A double transgenic mouse (mouse 4), Kras ^LSLG12D/+^ /p53 ^Fl/Fl^ was characterized by genotyping. **Kras** genotyping (left): Wild type (WT) = ~ 507 bp; Mutant = ~600 bp (Heterozygote = ~507 bp and ~ 600 bp); **p53** genotyping (right): Wild type = ~ 270 bp; Homozygote = ~ 390 bp (Heterozygote = ~ 270 bp and ~ 390 bp).

For the B6.129P2-*Trp53* ^tm1Brn^/J Trp 53 mouse strain, exons 2-10 of the Trp53 (transformation related protein 53) gene are flanked by *loxP* sites in this conditionally targeted mutation. Cre-mediated recombination deletes these exons, functionally abolishing p53 function [32]. However, prior to Cre-mediated recombination, the status of the p53 locus is normal. Therefore, we bred B6.129S4-*Kras* ^tm4Tyj^/J and B6.129P2-*Trp53* ^tm1Brn^/J transgenic mice to obtain a double transgenic Kras ^LSLG12D/+^. p53 ^fl/fl^ mouse as shown in Upper Figure 4, making it possible to simultaneously manipulate both p53 and Kras genes. Genotyping of five pups in the second breeding showed that we were able to obtain one Kras ^LSLG12D/+^. p53 ^fl/fl^ mouse (mouse 4 in Lower Figure 4). Because of the technical requirement for catheterization of the urinary bladder, only female mice were used in subsequent experiments.

### Soft tissue sarcoma formation after intravesical Adeno-Cre treatment of Kras ^LSLG12D/+^. p53 ^fl/fl^ female mice in the suture site

After intravesical instillation of Kras ^LSLG12D/+.^ p53 ^fl/fl^ female mice (6~8 weeks), we initially used a retention suture to hold the Adeno-Cre solution inside the bladder for 2 hours, as we had done in previous work [33]. A single suture was passed in a triangular fashion through three different points of the skin distal from the urethra to close it and retain the instillation fluid. After 2 hours, this suture was removed by cutting with scissors and pulling it through the non-knotted end, without further invasive animal manipulation. The intravesical instillation was applied only once in each mouse. Interestingly, after 4 to 8 weeks, a visible tumor appeared in five of five mice around the area of the original suture. No tumors occur for the wild type C57BL6 mice in the presence of Adeno-Cre.

**Figure 5:**
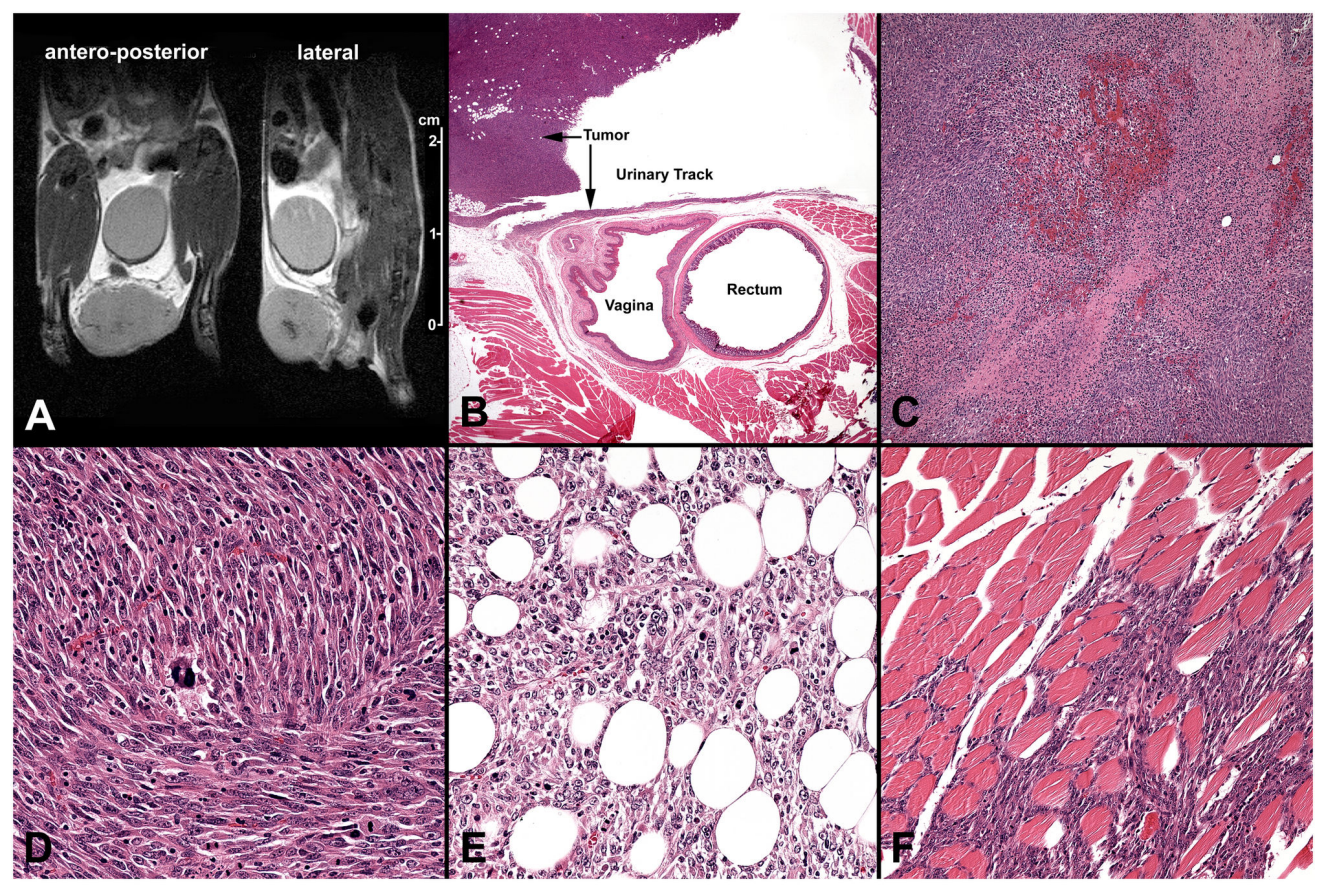
MRI (A) and histology (B-F) images from mice with Adeno-Cre treatment in the presence of sutures. Kras ^LSLG12D/+^ /p53 ^Fl/Fl^ Kras ^LSLG12D/+^ /p53 ^Fl/Fl^ mice were intravesically treated with 8.46×10^8^ pfu Adeno-Cre with a distal urethral suture placed for 2 hours, as described in Materials and Methods. When the pelvic tumors appeared (four to eight weeks after Adeno-Cre treatment), proton density-weighted MRI scans were performed and then the mice were sacrificed for histological examinations. **A**: MR images (coronal plane, left; sagittal plane, right) of lower body of mouse with lower pelvic tumor; **B**: Low power (2x) H&E section shows that the tumor is located in the pelvic soft tissue but not within the urinary track; sarcoma tumor with focal necrosis is apparent in **C** (10x); increased mitotic rate is apparent in **D** (40x); invasion of adipose tissue is apparent in **E** (40x); and invasion of striated muscle **F** (40x).

To more precisely identify the anatomical location of these tumors, a proton density-weighted MRI study was performed in two of these mice, capturing real-time visualization of the tumor location and size (Figure 5A). To assess the histology of these tumors, the mice were sacrificed and H&E stains were performed. As shown in Figure 5B, the histological location of these tumors was confirmed to be outside of the urinary track. Additional pathological examinations of the tumor revealed focal necrosis (Figure 5C), a spindle cell morphology with a high mitotic rate (Figure 5D), fat invasion (Figure 5E) and striated muscle invasion (Figure 5F), consistent with an aggressive tumor behavior.

**Figure 6:**
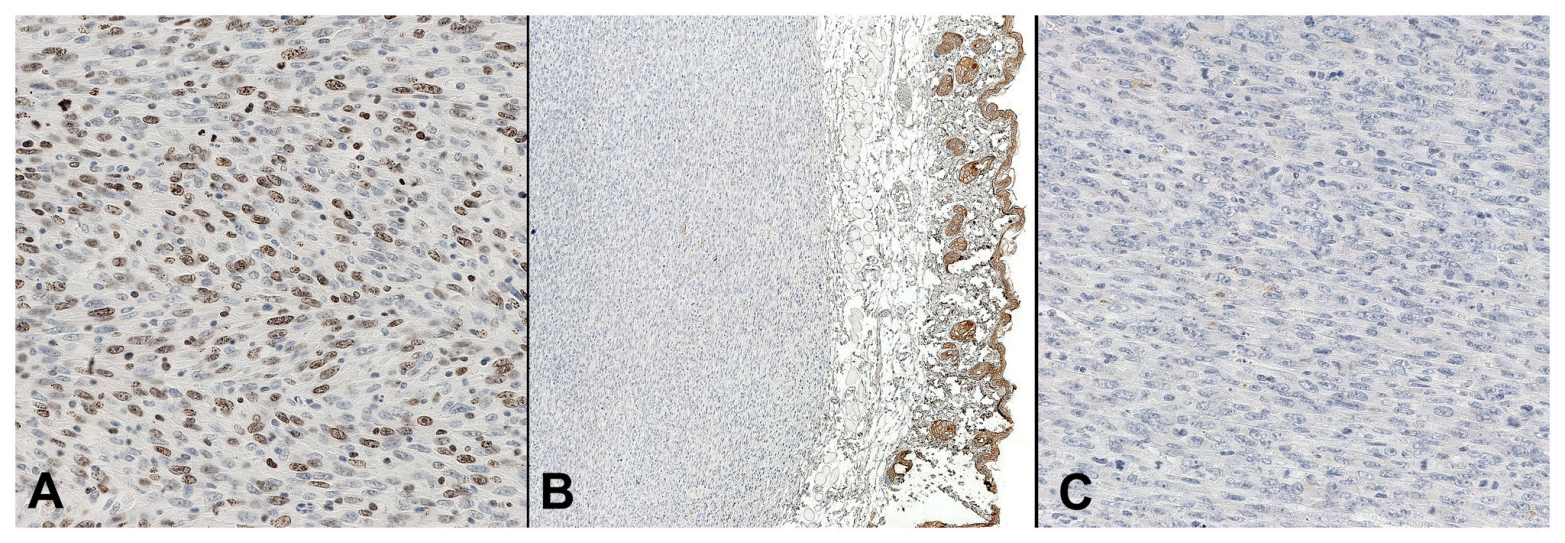
Immunohistochemistry of the pelvic soft-tissue sarcoma tumor induced in the Adeno-Cre treated Kras ^LSLG12D/+^ /p53 ^FL/FL^ mice. **A**: The high Ki67 expression in the nuclei of >70% of the tumor cells demonstrates a high proliferative rate (40x); Sarcoma tumor **B** (10x); **C** (40x) shows negative staining for pan-cytokeratin, demonstrating a non-epithelial phenotype; in **B**, only the skin layer is positive, acting as an internal positive stain control.

To further characterize the tumor type, immunohistochemistry was performed for Ki-67 pan-cytokeratin and p63 expression. As shown in Figure 6A-C, the tumor was Ki-67 positive in >70% of the tumor cell nuclei (Figure 6A), which correlates with a highly aggressive tumor. Pan-cytokeratin (Figure 6B, 6C) and p63 (data not shown) staining was negative, ruling out an epithelial origin of this tumor. Thus, based on tumor location, histological features and immunochemistry analysis, we concluded that this was a soft tissue sarcoma [34].

### Urothelial hyperplasia after intravesical Adeno-Cre treatment in Kras ^LSLG12D/+^. p53 ^fl/fl^ female mice with no urethral suture

Based on the above results of non-urinary tumors at the suture site, we modified the intravesical delivery of Adeno-Cre to eliminate the use of retention sutures. To guarantee an adequate amount of Adeno-Cre effect after the instillation we kept the delivering catheter inside the bladder for at least 3 hours in anesthetized mice and we repeated the instillation once per week for a total of four weeks. After treating these mice, we kept them in long-term observation and 4 to 6 months later they showed no evidence of tumor development.

**Figure 7:**
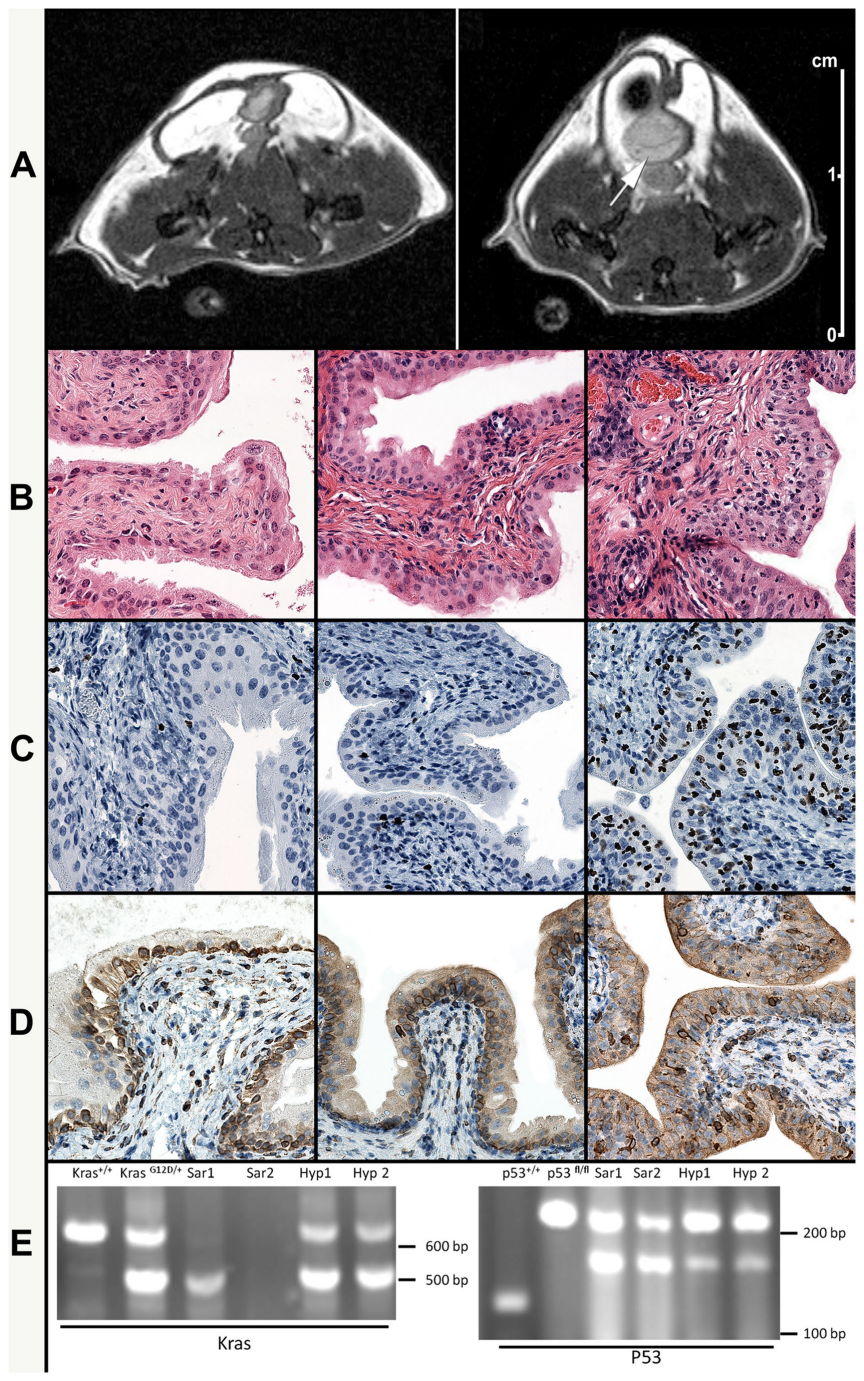
Induction of urothelial hyperplasia. Kras ^LSLG12D/+^ /p53 ^Fl/Fl^ mice were intravesically treated with Adeno-Cre (8.46×10^8^ pfu Adeno-Cre with 3-hour holding of catheter once per week for a total of four weeks and with no suture applied) 4.5 to 6 months after last instillation. Proton density-weighted MR images (axial plane) of the pelvis of a normal (control) mouse (**A**-**left**) and a 6-month treated mouse (**A-right**) showing focal thickening of the urinary bladder wall (arrow). As compared with histological images from a normal, control mouse (**left B, C and D**); the H&E histological sections show the presence of mild urothelial hyperplasia at 4.5 months (**B-center**) and moderate hyperplasia at 6 months (**B-right**). Ki67 staining shows no increase in nuclear expression in the bladder of a mouse after 4.5 months (**C-center**) but increased full thickness with nuclear Ki67 expression in the bladder urothelium after 6 months (**C**-**right**). COX-2 staining shows a modest increase in the cytoplasmic and peri-nuclear expression in the bladder urothelium at 4.5 months (**D-center**) and a moderate increase at 6 months (**D-right**). Recombination analysis of Kras and p53 genes was performed with both Kras (**E-left**) and p53 (**E-right**): Lane 1: wild type mouse; lane 2: Kras ^LSLG12D/+^ /p53 ^Fl/Fl^ mouse before Adeno-Cre treatment; lanes 3 and 4: sarcoma tissue from Kras ^LSLG12D/+^ /p53 ^Fl/Fl^ mice after Adeno-Cre treatment with suture (Sar1 and Sar2); lanes 5 and 6: bladder tissues from Kras ^LSLG12D/+^ /p53 ^Fl/Fl^ mice after Adeno-Cre treatment without suture (Hyp1 and Hyp2).

We also utilized MR imaging to complement the external monitoring of tumor growth. As shown in Figure 7A, thickening of urinary bladder mucosa appeared to develop in the treated double transgenic mice (right, Figure 7A) as compared to the bladder of normal mice (left, Figure 7A).

Histological examination was performed in two representative mice 4.5 and 6 months after the last instillation. We observed urothelial hyperplasia (center and right, Figure 7B) as compared with the bladder of normal mice (left, Figure 7B). To characterize this hyperplasia, immunohistochemistry stains for Ki67 and COX-2 were performed. Ki67 is one of well-known biomarkers for the study of cell proliferation and has been frequently used in mouse models [18,35]. COX-2 is an inflammation index marker and has been found highly expressed in bladder cancer [36] and related to Kras activation ( [37]. As shown in Figure 7C-right, Ki67 positive nuclear staining was observed in ~50% of the cells from the hyperplastic urothelium of the mouse sacrificed at 6 months after treatment, in contrast to the <10% positive nuclear staining of the normal, control mice (left, Figure 7C). However, a milder (~10-20%) increase in Ki67 expression was observed in the urothelium of the mice sacrificed at 4.5 months (center, Figure 7C). The majority of the proliferating cells in the normal (control) mice were located in the lower, most basal cell layer of the urothelium. However, the Ki67 expression in the mouse at 6-month after treatment was observed in the full thickness of the urothelium. No evidence of malignant invasion was observed in any of the animals studied.

As compared with a normal, control mouse (left, Figure 7D), COX-2 staining showed a moderated increase (2+) in the cytoplasmic expression in the bladder urothelium of a double transgenic mouse at 6 months post Adeno-Cre intravesical treatment (right, Figure 7D) while a milder increase (1+) of COX-2 was observed in a double transgenic mouse at 4.5 months post treatment (center, Figure 7D).

Finally, to detect the status of Kras and p53 in both sarcoma tumors and hyperplastic bladder tissue, PCR analysis was performed. Figure 7E shows that both Kras and p53 were recombined (Kras was activated and p53 was inactivated) but the sarcoma tissues had a higher recombination ratio than the hyperplastic bladder tissues. Comparing our findings to available standards (http://web.mit.edu/jacks-lab/protocols_table.html), our PCR results for Kras primers did not yield any fragment at 650 bp in the recombinated tissues. Instead, tissues from Kras^G12D/+^ mice without Adeno-Cre treatment exhibited two fragments at 500 bp and 620 bp while the fragment at 620 bp, or both fragments at 500 bp and 620 bp, disappeared in the sarcoma tissues from our Adeno-Cre treated Kras^G12D/+^ mice. However, tissues from hyperplastic bladders exhibited a reduced recombination Kras ratio in comparison with the sarcoma tissues (Figure 7E).

For analysis of p53 status, PCR of wild type mouse tissue yielded a fragment at 130 bp while PCR of p53 floxed/floxed, unrecombined alleles yielded a 212 bp fragment. In the sarcoma tissues we found a 168 bp fragment of p53 floxed/floxed, recombined alleles with inactivation of p53. The presence of both 168 bp and 212 bp fragments in the sarcoma tissues indicated that the recombination of p53 floxed/floxed is not 100% complete, most likely due to the mixture of tumor and normal cells. Similar to the Kras status, tissues from hyperplastic bladders exhibited a reduced p53 recombination ratio as compared with the sarcoma tissues (Figure 7E). The results of the p53 recombination analysis are consistent with Dr. Berns lab’s [38] and support the successful specific Cre recombination using this *in vivo* delivery route.

The numbers of animals with controls and experiments, performed MRI, IHC and PCR are listed in Table 1.

**Table 1:** Summary of animal numbers of sarcoma formation and hyperplasia.

	Cohort size	Development of sarcoma	Development of hyperplasia	Assessed by MRI	Histologic evaluation (H&E, pan-cytokeratin, COX-2, Ki67, p63 - respectively)	Mice with confirmed Kras and p53 status
Transgenic mice treated with Adeno-Cre in the presence of suture	5	5	N/A	**3**	Sarcoma (3, 3, 0, 3, 3)	4
Transgenic mice treated with Adeno-Cre In the absence of suture; 4.5 months	4	0	Moderate, 4	0	Bladder (4, 0, 2, 2, 0)	4
Transgenic mice treated with Adeno-Cre In the absence of suture, 6 months	4	0	High, 4	2	Bladder (4, 0, 2, 2, 0)	4
C57BL6 wild typeice treated with Adeno-Cre as control	4	0	0	2	Bladder (4, 0, 3, 3, 0)	2

## Materials and Methods

### Mice

B6.129S4-*Gt*(*ROSA*) *26Sor* ^tmlSor^/J (#003474), B6.129S4-*Kras* ^tm4Tyj^/J (#008179), B6.129P2-*Trp53* ^tm1Brn^/J (#008462), and C57BL/6J (#000664) mice were purchased from Jackson Laboratory (Bar Harbor, ME, USA). B6.129S4-*Kras* ^tm4Tyj^/J mice were crossed to B6.129P2-*Trp53* ^tm1Brn^/J mice according to the scheme in Figure 4A. This study was carried out in strict accordance with the recommendations in the Guide for the Care and Use of Laboratory Animals of the National Institutes of Health. The animal protocol was reviewed and approved by the Institutional Animal Care and Use Committee of the University of Colorado Anschutz Medical Campus (Approved Number: B-9111(03) 1E). All surgery was performed under sodium pentobarbital anesthesia and all efforts were made to minimize suffering.

### Chemicals

Potassium ferricyanide crystalline, potassium ferricyanide trihydrate and magnesium chloride were purchased from Sigma Aldrich (St. Louis, MO, USA).

### Adenovirus-Cre Vector Preparation

Adeno-Cre was either obtained from Gene Transfer Vector Core, University of Iowa (Iowa City, IA) or home- made. The commercial available Adeno-Cre was made based on the reference [39] while the preparation of home-made Adeno-Cre was based on the process following: Adenovirus-Cre was grown in large scale in HEK 293 cells, purified by CsCl gradient centrifugation as previously described [40] and dialyzed into optimized storage buffer [41] modified by use of 50% v/v glycerol in place of 5% w/v sucrose. The virus concentration was determined by OD_260_, with 1 OD_260_ unit equal to 10^10^ infectious viral particles/ml.

### Adeno-Cre instillation

Treatment with Adeno-Cre in the presence of the suture was performed following a previous-described procedure [33]. Female C57/BL6J, ROSA or Kras ^LSLG12D/+^. p53 ^fl/fl^ mice 6 to 8 wk of age were used and either 8.46×10^8^ or 4.23×10^8^ pfu Adeno-Cre or 8.46×10^8^ pfu Adeno-empty virus per mouse were applied.

### X-Gal Staining

The urinary bladder, tumors and other organ tissues were harvested at days 3, 7, 14 and 28 respectively post Adeno-Cre intravesical instillation and snap-froze with OCT (Sakura Fineteck USA, Inc, Torrance, CA, USA). The frozen blocks were stored at -80°C for further processing. The blocks were freshly cut into 5 µM sections and stained with X-galactosidase solution as described [42]. Microscopic pictures were taken using a Nikon ECLIPSE TE2000-S microscope.

### Genotyping of mice and PCR analysis of recombination

Genotyping of mice and PCR analysis of recombination of the mouse strain were performed using protocols from Jackson Laboratory website (http://web.mit.edu/jacks-lab/protocols_table.html) and [38] with minor modifications.

### Histological analysis, immunofluorescent staining and immunohistochemistry

Freshly dissected tissues were bisected, fixed in 10% buffered formalin and embedded in paraffin. Sections were stained using hematoxylin and eosin (H&E), followed by histopathologic examination. Immunofluorescent staining was performed as previously described [43]. Primary antibodies used were: anti-β-galactosidase (Rockland rabbit polyclonal, 200-4136; 1:500) and anti-Cytokeratin 7 (Abcam mouse monoclonal, ab9021; 1:50). Secondary antibodies were rabbit anti-mouse IgG (H+L) # A-21427 (Invitrogen/Molecular Probes Carlsbad, California AlexFluor 555) or goat anti-rabbit IgG (H+L) # A-11008 (AlexFluor 488) (both at 1:200 in TBST). Immunohistochemistry was performed according to conventional procedures using an antibody for Ki-67 (Neomarkers/Thermo Scientific, Waltham, MA; rabbit monoclonal SP6; cat# RM-9106-SO; dilution 1:300 in TBST + 1% BSA w/v) and an antibody for COX-2 (Biocare Medical, Concord, CA; rabbit monoclonal SP21; cat# CRM306A; dilution 1:100 in TBST + 1% BSA w/v).

For p63 staining, p63 (Santa Cruz Biotechnology, Santa Cruz, CA; mouse monoclonal 4A4; cat#: sc-8431; Lot: K1908; 1:200; diluted in TBST + 1% BSA w/v + 0.05% ProClin 950). Antigens to p63 were revealed in 10 mM sodium citrate pH 6.0 solution for 5 minutes at 125°C (22 psi; Decloaking chamber, Biocare) with a 10 minute ambient cool down. Immunodetection was performed on the NexES autostainer (Ventana Medical Systems, Tucson, AZ) at an operating temperature of 37°C. An Option dispenser was added to the run, containing Mouse Ig blocking reagent (Vector Labs, Burlingame, CA; cat#: MKB-2213; diluted 1:10 in PBS pH 7.6; 30 minute incubation). The primary antibody was incubated for 30 minutes and detected with a modified I-VIEW universal DAB kit (Ventana). The secondary antibody and SA-HRP dispensers were each replaced with full strength Mouse on Mouse ImmPress reagent (Vector Labs, Burlingame, CA; cat#: MP-2400; 8 minute incubation each dispenser). Sections were counterstained in Harris hematoxylin for 2 minutes, blued in 1% ammonium hydroxide (v/v), dehydrated in graded alcohols, cleared in xylene and coverglass mounted using synthetic resin.

Stains were performed by a certified histotechnologist (author EEG) and the slides were reviewed by a certified pathologist (author FLR).

### Magnetic Resonance Imaging (MRI)

Animals were anesthetized with 2% isoflurane and placed onto a mouse holder. A Bruker 4.7 Tesla/ 16-cm MRI/MRS PharmaScan (Bruker Medical, Billerica, MA) with a mouse volume transmitter/ receiver coil (36 mm diameter, tuned to 200 MHz for 1H) was used for all MRI studies. After obtaining localizer images (fast MRI tri-pilot), fast spin echo RARE (rapid acquisition with relaxation enhancement) proton density (PD)-weighted MRI scans were obtained in three planes (axial, coronal and sagittal) for bladder localization. The scan parameters were: FOV=4.00 cm; slice thickness 1 mm; inter-slice distance 1 mm (no gap between slices); TE/TR=30/3000 ms; number of slices 16; number of averages 2; flip angle 180 degrees; matrix size 256x256; total acquisition time 3min 17sec (per plane). No gadolinium contrast agent was used. All images were processed using Bruker ParaVision software (Version 4.1.0).

## Discussion

Urothelial carcinoma is a common human cancer, and ranks as one of the most costly cancer types to treat due to its frequent recurrence [44]. Compared with other cancers, animal modeling in bladder cancer is under-developed [45]. Personalized cancer medicine, relying on targeted therapy, is emerging and aiming for a customized treatment rather than a generic disease-based approach [46]. Thus, a clinically relevant animal model is needed for the rapid evaluation of any promising treatment for bladder cancer.

Several *in vivo* animal models for various cancers have been developed. Subcutaneous (SC) flank xenograft models are commonly used in various cancer types because it is easy to establish, convenient to manage and lends itself to ready quantification of the tumor burden. The main disadvantage of this approach in bladder cancer is the loss of the compartmental effect of treating superficial bladder cancer when establishing subcutaneous bladder tumors that are not orthotopic. In contrast to the SC flank model, orthotopic human tumor cell implantation simulates the natural environment of bladder cancer, with intact pathological responses, making this model the closest to the clinical setting and the most applicable in bladder cancer research [47] [33]. However, this model has several limitations such as the inability to mimic the gene mutation status or the real development of human bladder cancer. In addition, such models required the use of immunosuppressed animals to avoid xenograft rejection and allow proper tumor implantation. The results collected from immunodeficient animals always have a very limited extrapolation to immunocompetent human cancer settings.

In fact, an ideal murine tumor model should closely mimic human-related tumorigenesis in terms of genetic and physiological conditions [48]. To achieve this goal, genetically manipulated mouse models have been created [48]. One of the most attractive features of these models is the ability to manipulate genes in a tissue-specific, time-controlled fashion. Transgenic models of bladder carcinogenesis, using the uroplakin II gene (UPII) to drive urothelium specific expression of SV40T antigen crossed with a *cre* transgenic strain model, developed by Dr. Wu and his coworkers, has been described [17,18,25,49]. Cre recombination using Adeno-Cre offers a special type of site-specific recombination and has been used to study many cancer types including: lung cancer [2], breast cancer [50] and head and neck squamous cell carcinomas [51]. Recently, a two hit (Kras activation and p53 inactivation) transgenic mice model has been reported to accelerate formation of oral and lung cancers [1,21].

Using a laparotomy technique for the delivery of Adeno-Cre, another group has reported gene deletion exclusively in the epithelium of the bladder [29] by inducing "invasive bladder cancer." However, our study demonstrates that the tumor formed using these similar surgical techniques is unlikely to be from urothelial origin but an artifact due to the leak of Adeno-Cre into the surrounding soft tissue inducing soft-tissue sarcomas. A larger dose of Adeno-Cre (1×10^10^ pfu) than that used by Dr. Jacks group in a lung cancer model (2.5×10^7^ pfu) was used [1]. It is reasonable that a larger dose of Adeno-Cre may be needed in the urinary bladder since unlike intratracheally administrated Adeno-Cre in the lungs, the elimination of urine could dilute or remove by “flushing” some of the Adeno-Cre delivered to the bladder cavity. Due to application of Adeno-Cre fluid retention in our study, the dose of Adeno-Cre is less than that used by the Dr. Cory Abate-Shen group [29]. Importantly, our study adds significant information by defining the temporal and peak effect of intravesical Adeno-Cre’s action on the urothelium via the ROSA26 experiments, which will be important if future applications.

Our results showed that intravesical delivery of Adeno-Cre into the bladder cavity of double transgenic Kras ^LSLG12D/+^. p53^fl/fl^ mice induced urothelial hyperplasia but did not cause bladder carcinogenesis during a maximum observation period of 6 months. Unexpectedly, a spindle-cell tumor consistent with sarcoma developed quickly when a suture was used in our first experiments with double transgenic mice. This observation is in accordance with a previous study that direct delivery of Adeno-Cre into soft tissue of Kras ^LSLG12D/+^ p53 ^fl/fl^ mice induces sarcoma formation [34]. At first glance, this is very surprising since we intentionally did not deliver Adeno-Cre outside of the urinary track. However, five double transgenic mice treated with Adeno-Cre in the presence of suture developed sarcoma tumors, likely due to the unavoidable soft tissue exposure to the instillation fluid at the suture site. Soft tissue sarcomas may arise from skeletal muscle (rhabdomyosarcomas), adipose tissue (liposarcomas) and fibroblasts (fibrosarcomas). Based on the histological features of the observed tumors, the diagnosis of fibrosarcoma is most likely, warranting further investigation. The quick development of a sarcoma-like tumor as compared to the slow formation of urothelial hyperplasia is notable and may be explained by the importance of different microenvironments on cancer development [52]. For example, striated muscle cells do not divide and urothelial epithelial cells have a constant turn over. In addition, urothelial cells are constantly exposed to urine, which usually carries higher concentrations of toxic substances as compared with blood concentrations. This would make the urothelial lining to express a distinct defensive phenotype to reduce the reabsorption of such toxic substances from the urine, both within the urothelial cells and back into circulation. Furthermore, this protective function may be even present at the genetic level making the urothelium less susceptible to carcinogenesis. Interestingly enough, muscle stem cells exhibit robust regenerative capacity in vivo [52] but urothelial epithelial cells have a very slow turnover rate [53].

Furthermore, Ki67 immunohistochemistry stain of hyperplastic bladder (Figure 7C) revealed a large number (>70%) of urothelial cells expressing this protein, suggesting that Adeno-Cre largely transduced these cells. Protein analysis of COX-2 by immunohistochemistry revealed that this inactivation of p53 and activation of Kras induced an anti-inflammatory reaction (Figure 7D). Thus, regardless of the induced genetic alterations, these urothelial cells did not develop carcinogenesis but responded only with hyperplastic changes.

Nevertheless, we likely did not achieve either complete inactivation of p53 or absolute activation of Kras in bladder based on recombination analysis (Figure 7E). The complete loss of p53 may be required in addition to activation of oncogenesis in order to induce tumorigenesis, which may also explain why we did not see solid tumors in the bladder [13]. Additionally, carcinogenesis may require more than the 6 month maximum time observation used in this study. In support of this possibility, we saw a trend towards increase cell proliferation at 6 months (Figure 7C).

Interestingly, results from other studies demonstrated that the combinations of p53 with pRb [54] and Kras with β-catenin [18] were sufficient to drive bladder tumorigenesis. Supporting our proposed microenvironment hypothesis, a recent study from the same research group [17] using mice with Kras or β-catenin and FGFR-3 mutations plus the uroplakin II Cre promoter, demonstrated expontaneous induction of tumorigenesis in the skin (UroIICre^+^ Fgfr ^3+/K644E^ Kras ^G12D/+^ mice) and lung skin (UroIICre^+^ Fgfr ^3+/K644E^ β-catenin ^exon3/+^ mice) but not in the urinary bladder when animals were 1 year old.

Although Cre/LoxP models provide an excellent means to control genetic events at specific endogenous loci, they are limited by the difficulty of introducing Cre into a cancer-initiating cell [48]. However, in the present study, we have successfully obtained simultaneous manipulation of both Kras and p53 expression in vivo as attested by the development of soft tissue sarcoma tumors and urothelial hyperplasia (Figure 7E). Nevertheless, there are several limitations in this study. One disadvantage is that a relative large amount of Adeno-Cre is required [29]. A second drawback is that only female mice could be used, as the urinary catheter cannot be passed through the murine penis. The use of male mice might be more clinically relevant since bladder cancer occurs more frequently in males than in female.

In summary, we have demonstrated a new murine model of urothelial hyperplasia with several advantages over existing bladder cancer models. The ability to use direct Adeno-Cre urinary bladder instillation may be useful in testing the roles of other oncogenes and tumor suppressors in this setting and is less invasive the other described method of Adeno-Cre delivery for urothelial-specific activation.
